# Neuromodulation Enables Temperature Robustness and Coupling Between Fast and Slow Oscillator Circuits

**DOI:** 10.3389/fncel.2022.849160

**Published:** 2022-03-28

**Authors:** Carola Städele, Wolfgang Stein

**Affiliations:** School of Biological Sciences, Illinois State University, Normal, IL, United States

**Keywords:** stomatogastric ganglion (STG), neuromodulation, temperature, coupling, robustness, gastric, central pattern generator, peptide

## Abstract

Acute temperature changes can disrupt neuronal activity and coordination with severe consequences for animal behavior and survival. Nonetheless, two rhythmic neuronal circuits in the crustacean stomatogastric ganglion (STG) and their coordination are maintained across a broad temperature range. However, it remains unclear how this temperature robustness is achieved. Here, we dissociate temperature effects on the rhythm generating circuits from those on upstream ganglia. We demonstrate that heat-activated factors extrinsic to the rhythm generators are essential to the slow gastric mill rhythm’s temperature robustness and contribute to the temperature response of the fast pyloric rhythm. The gastric mill rhythm crashed when its rhythm generator in the STG was heated. It was restored when upstream ganglia were heated and temperature-matched to the STG. This also increased the activity of the peptidergic modulatory projection neuron (MCN1), which innervates the gastric mill circuit. Correspondingly, MCN1’s neuropeptide transmitter stabilized the rhythm and maintained it over a broad temperature range. Extrinsic neuromodulation is thus essential for the oscillatory circuits in the STG and enables neural circuits to maintain function in temperature-compromised conditions. In contrast, integer coupling between pyloric and gastric mill rhythms was independent of whether extrinsic inputs and STG pattern generators were temperature-matched or not, demonstrating that the temperature robustness of the coupling is enabled by properties intrinsic to the rhythm generators. However, at near-crash temperature, integer coupling was maintained only in some animals while it was absent in others. This was true despite regular rhythmic activity in all animals, supporting that degenerate circuit properties result in idiosyncratic responses to environmental challenges.

## Introduction

Acute temperature changes are a significant challenge to neurons and neuronal circuits because they alter active and passive biophysical membrane properties but rarely linearly or equally across properties (Hille, 1978; Collins and Rojas, 1982; Cao and Oertel, 2005). Consequently, temperature changes can disrupt neuronal activity with severe consequences for behavior and survival. All levels of neuronal processing are affected by temperature changes, including active and passive properties, dendritic and somatic integration, and synaptic release and neuronal coupling. Possessing compensatory mechanisms that protect neurons from detrimental temperature changes are thus vital components of survival. Such compensatory mechanisms are particularly relevant for poikilothermic animals, which can experience significant environmental temperature fluctuations on different time scales (from rapid and intermittent changes to slow and permanent ones).

**FIGURE 1:**
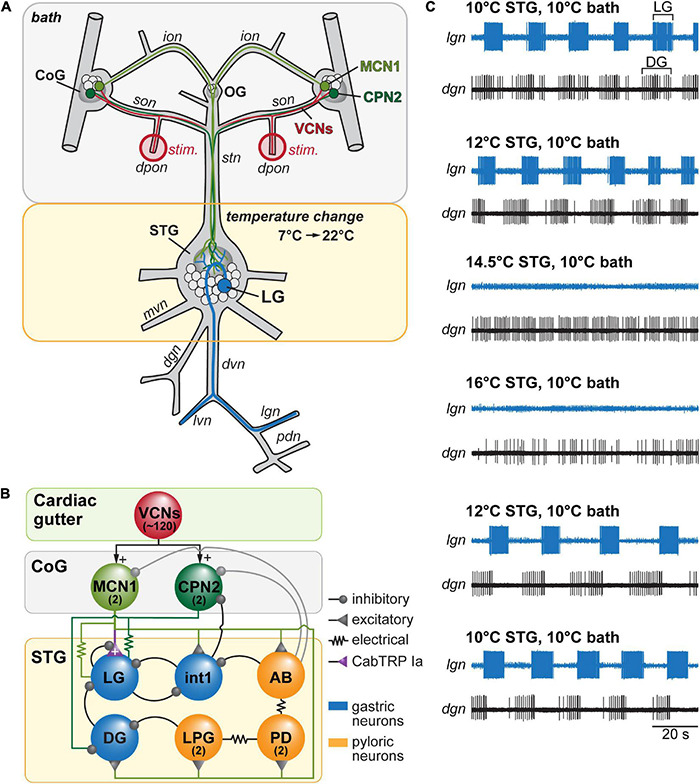
Spontaneously active gastric mill rhythms crash when only the stomatogastric ganglion is heated. **(A)** Schematic of the stomatogastric nervous system (STNS) illustrating the experimental approach. A split-bath approach was used to selectively heat either the stomatogastric ganglion (STG, yellow), or its extrinsic inputs including the esophageal ganglion (OG) and the commissural ganglia (CoGs; bath, gray), which house the descending projection neurons MCN1 (modulatory projection neuron 1) and CPN2 (commissural projection neuron 2). Gastric mill activity was assessed by recording the lateral gastric (LG) neuron (blue) extracellularly on the lateral gastric nerve (*lgn*). Except for the experiments shown in **(C)**, gastric mill rhythms were initiated by activating the ventral cardiac neurons (VCNs) through stimulation of the paired dorsal posterior oesophageal nerves (*dpons*) (‘stim.’, red). VCN activity leads to a long-lasting activity of MCN1 and CPN2 (Beenhakker and Nusbaum, 2004). **(B)** Simplified wiring diagram. Neuron copy numbers are in parentheses. For complete circuit diagram see Powell et al. (2021). VCN activity leads to a long-term excitation of MCN1 and CPN2, which in turn innervate the gastric mill and pyloric pattern generators. LG receives excitatory input from MCN1 via release of Cancer borealis tachykinin-related peptide Ia (CabTRP Ia). Gastric mill neurons: LG, Interneuron 1 (Int1), dorsal gastric neuron (DG). Pyloric neurons: anterior burster (AB), pyloric dilator (PD), lateral posterior gastric (LPG). AB is the main pacemaker driving the pyloric rhythm. **(C)** Example of a crash of spontaneous gastric mill activity at elevated temperature. Shown are extracellular recordings of the *lgn* and *dgn* (dorsal gastric nerve) at 10, 12, 14.5, and 16°C STG temperature. LG is the only large-amplitude neuron on the *lgn*. Similarly, DG is the only large-amplitude neuron on the *dgn*. Outside bath temperature was kept constant at 10°C. The rhythm failed when STG temperature was elevated to 14.5 or 16°C, but could be recovered by cooling the STG back to 12°C, and 10°C. *Further abbreviations:* PD, pyloric dilator neuron; *ion*, inferior esophageal nerve; *son*, superior esophageal nerve; *stn*, stomatogastric nerve; *mvn*, medial ventricular nerve; *dvn*, dorsal ventricular nerve; *lvn*, lateral ventricular nerve; *pdn*, pyloric dilator nerve.

The coordination of the activities of coupled neural networks at different temperatures is particularly challenging, but often required for proper behavioral functioning. Powell et al. (2021) have recently demonstrated that the coordination between two oscillatory circuits in the stomatogastric nervous system (STNS, Figure 1A) of the crab *Cancer borealis* is maintained across a broad range of temperatures (7–23°C). The gastric mill and pyloric central pattern generators (CPGs) are coupled, but these rhythms run at very different speeds (Nadim et al., 1998; Bartos et al., 1999; Stein, 2017). Together, these CPGs orchestrate food processing in the crab foregut. The episodic gastric mill rhythm has a cycle period of ∼10 s and controls the slow movement of three teeth in the gastric mill chamber of the foregut. The continuous pyloric rhythm is about ten times faster (∼1 s) and controls the filtering of chewed food particles through the pylorus into the midgut. Both rhythms, as well as their coordination, are considered essential for the survival of the animal. *In vivo*, after food intake or stimulation of sensory pathways involved in feeding, the gastric mill rhythm is activated, and both rhythms are active together (Diehl et al., 2013; Yarger and Stein, 2015). Similarly, stimulation of the same sensory pathways in the isolated nervous system (*in vitro*) leads to a long-lasting gastric mill rhythm so that both rhythms are active simultaneously (Beenhakker et al., 2004). In this condition, the gastric mill cycle period is an integer multiple of the pyloric cycle period (= integer coupled; Powell et al., 2021). This integer coupling is mainly mediated by a single synaptic connection that provides pyloric-timed inhibition to the gastric mill pattern generator (Nadim et al., 1998; Bartos et al., 1999).

The temperature responses of the two rhythms are well characterized. The pyloric rhythm is remarkably robust and remains functional over a range of more than 20°C (Tang et al., 2010; Soofi et al., 2014; Haddad and Marder, 2018), both *in vitro* and *in vivo*. Computational models predict that the temperature robustness of the pyloric rhythm is intrinsic to the CPG circuit itself (O’Leary and Marder, 2016; Alonso and Marder, 2020). Experimental approaches support these predictions, showing that even when only the stomatogastric ganglion, home to the pyloric CPG, is heated, the pyloric rhythm retains its broad temperature robustness.

At first glance, the temperature response of the episodic gastric mill rhythm seems very different because this rhythm crashes and stops when the stomatogastric ganglion with the gastric mill CPG is warmed by only a few degrees Celsius (Städele et al., 2015). However, it has been long known that neuromodulatory pathways extrinsic to its CPG are critical for initiating and maintaining the gastric mill rhythm. Specifically, a diverse set of sensory pathways, including chemosensory, proprioceptive and mechanosensory, excite the commissural ganglia’s modulatory projection neurons (Stein, 2009). These projection neurons extend long axons to the stomatogastric ganglion where the gastric mill and pyloric CPGs are located. In the stomatogastric ganglion, the projection neurons release a diverse set of neuropeptides that activate the gastric mill circuits (Marder, 2012).

It is also clear that such extrinsic neuromodulatory input can sustain the gastric mill rhythm at elevated temperatures. We have demonstrated that increased neuropeptide release from descending modulatory projection neurons rescues the gastric mill rhythm from a crash at elevated temperature, and that projection neuron activity can increase with temperature (Städele et al., 2015; DeMaegd and Stein, 2021). It has also been demonstrated that in intact animals, where temperature changes affect both, CPG circuits and their extrinsic modulatory inputs, the gastric mill rhythm is sustained at elevated temperatures. It is thus conceivable that extrinsic neuromodulation is a mechanism to bestow temperature robustness to neural oscillators. However, the temperature range over which such mechanism may function remains unclear. Powell et al. (2021) have recently demonstrated that the gastric mill rhythm can indeed be similarly temperature-robust as the pyloric rhythm (a range of >20°C) even in the isolated nervous system. In addition, the integer coupling between the gastric and pyloric rhythms seems to prevail at elevated temperatures as well. However, several questions remain. Notably, the experiments by Powell et al. (2021) could not determine whether descending modulatory neurons extrinsic to the gastric mill CPG enable the temperature robustness of the gastric mill rhythm or if the gastric mill CPG, like the pyloric CPG, is bestowed with intrinsic temperature robustness. Furthermore, whether modulatory projection neurons enable or influence the gastric mill integer coupling across temperature is unknown. Our study separated temperature effects on extrinsic projection neurons from those on the gastric mill and pyloric CPGs in the STG. We achieved this by selectively and differentially changing the temperature of the STG and the projection neurons. This enabled us to provide a mechanism for the temperature robustness of the gastric mill rhythm and to determine the impact of extrinsic neuropeptide modulation on the integer coupling observed by Powell et al. (2021).

## Materials and Methods

### Animals

Adult *Cancer borealis* were purchased from The Fresh Lobster Company (Gloucester, MA, United States) and maintained in filtered, aerated artificial seawater (salt content ∼1.024 g/cm^3^, Instant Ocean Sea Salt Mix, Blacksburg, VA, United States) at 10–11°C with a 12-h light-dark cycle. Animals were kept in tanks for at least 10 days prior to experiments.

### Solutions

*Cancer borealis* saline was composed of (in mM) 440 NaCl, 26 MgCl_2_, 13 CaCl_2_, 11 KCl, 11 Trisma base, and 5 maleic acid, pH 7.4–7.6 (Sigma Aldrich). In some experiments, 1 μM CabTRP Ia (GenScript, Piscataway, NJ, United States) was added to the saline. Solutions were prepared from concentrated stock solutions immediately before the experiment. Stock solutions were stored at −20°C in small quantities. Measurements were taken after 45 min wash in/out.

### Dissection

Animals were anesthetized on ice for at least 30 min. The stomatogastric nervous system was dissected following standard procedures as described previously (Städele et al., 2015) and pinned out in a silicone-lined petri dish (Sylgard 184, Dow Corning). Preparations were continuously superfused with physiological saline (7–12 mL/min).

### Temperature Control

A petroleum jelly well was built around the STG to thermally isolate it from the rest of the nervous system (Figure 1A). Temperature inside and outside the well was controlled independently with two saline superfusion lines, each heated by separate Peltier devices. Temperature was altered between 7 to max. 25°C in 3°C increments and changed by ∼1°C/min ± 0.2°C. The preparations were allowed to acclimate to the new temperature for 5 min before gastric mill rhythms were elicited between each temperature step. Temperature was continuously measured close to the STG and commissural ganglia with separate temperature probes (Voltcraft 300K, Conrad Electronic, Germany). Saline inflow to the nervous system was positioned within 1 cm of the STG so that the measured temperature at the point of inflow was approximately that of the ganglion somata. Three different temperature alternation experiments were performed. (1) Temperature in the STG well was altered, while the surrounding nervous system was kept constant at 9°C. (2) Temperature of the surrounding nervous system was changed (= bath), while the STG circuits were continuously kept at 10°C. (3) Temperature of both the STG circuits and the surrounding nervous system was altered simultaneously.

### Electrophysiological Recordings

Extracellular recordings were performed from nerves via stainless steel pin electrodes procedures (Städele et al., 2017). Stretches of the *lgn*, *dgn*, *mvn*, *pdn*, and *ions* were electrically isolated from the bath with Vaseline wells. Voltage signals were recorded, amplified, and filtered using an AM Systems amplifier (Model 1700; Everett, WA, United States) and digitized at 10 kHz with a Power 1401 (CED, Cambridge, United Kingdom).

### Gastric Mill Rhythm Stimulation

To elicit a gastric mill rhythm, we bilaterally stimulated the dorsal posterior esophageal nerves (*dpons*). Extracellular stimulation was performed using standard procedures (Städele et al., 2017) using a Master-8 stimulator (AMPI, Jerusalem, Israel) with 10 stimulus trains of 6 s duration at 0.6 Hz. Intratrain stimulation frequency was 15 Hz and each stimulus pulse was 1ms in duration (Beenhakker et al., 2004). The stimulation amplitude was set to 1.5 times the minimum voltage needed to elicit a response of STG neurons. The stimulus site was maintained at constant temperature throughout the whole experiment.

### Data Analysis, Statistical Analysis

Electrophysiological files were recorded, saved, and analyzed using Spike2 (Version 7.18, CED, Cambridge, United Kingdom), and original Spike2 and Matlab scripts. No spike sorting was necessary as all neurons could be individually identified on the respective nerve recordings, or from intracellular recordings. In case of MCN1 recordings from the *ion*, the large action potentials of the esophageal motor neuron were removed prior to measuring MCN1 following established protocols (Hedrich et al., 2011).

Bursts in all neurons were defined using inter-spike intervals and spike numbers. Inter-spike intervals longer than 0.25 s were defined as a pause between bursts (inter-burst interval) of the pyloric dilator (PD) neuron. PD exists in two copies, both of which are part of the pyloric pacemaker ensemble. Inter-spike intervals longer than 1 s were defined as a pause between the bursts of LG and DG. For all neurons, a minimum of 2 spikes was required to define a burst. Cycle periods were calculated from the beginning of one burst to the beginning of the next (PD for pyloric cycle period, LG for gastric mill cycle period). Duty cycles were calculated by dividing the burst duration by cycle period.

All data was normally distributed, and we employed parametric tests (ANOVA, student’s *t*-test, Rayleigh *z* test). In cases where different variables were measured in individual animals, repeated measures tests were considered. The factors and specific designs of each ANOVA are described in the corresponding figure legends. Statistical tests were performed using SigmaStat (version 11; Systat Software, San Jose, CA, United States). For ANOVA, statistical tests are reported in the format: statistical test, *F*(degrees of freedom, residual) = *F* value, *p* value, *post hoc* test, number of experiments. “N” denotes the number of preparations, while “n” denotes the number of trials. *Post hoc* tests are at a significance level of 0.05. Data was prepared in Excel and MATLAB and finalized in Adobe Illustrator (version 25.4.1; Adobe Inc., San Jose, CA, United States). In figures, data is presented as mean ± SD unless otherwise noted. In some figures, average data are connected by a line when recorded in multiple conditions to indicate paired data.

### Integer Coupling Analysis

To determine whether integer coupling was present or not, we calculated the significant. A detailed description is given in Powell et al. (2021). In short, the significant is calculated by dividing the gastric mill cycle period by the pyloric cycle period, and then taking the decimal part of the result. When zero or one, integer coupling is present because an integer number of pyloric cycles is present in one gastric mill cycle.

As a second measure for integer coupling, we used the phase at which the LG bursts started within the pyloric cycle. Phase was calculated by first measuring the time delay at which the LG burst started after the last preceding PD burst. This time was then divided by the pyloric cycle period, resulting in a number between 0 and 1 (the onset phase of LG). In any given condition (e.g., at one temperature), LG onset phase must remain the same for each burst if integer coupling is present.

To determine if any set of significant or LG onset phases was significantly different from a uniform distribution, we created rose (circular) plots and carried out a Rayleigh *z* test (Ruxton, 2017). Significant and phases were plotted in a circle from 0 to 1, and mean directional vectors were calculated. Uniform distribution indicates the absence of integer coupling, a rejection of the null hypothesis rejects uniformity and supports integer coupling.

## Results

### Spontaneous Gastric Mill Rhythms Are Not Temperature Robust Without Neuromodulatory Compensation

The stomatogastric ganglion (STG, Figure 1A) houses the gastric mill and pyloric central pattern generating networks. The episodic gastric mill rhythm requires activation through descending projection neurons (Stein, 2009). Cell bodies of most projection neurons that innervate the STG (∼20 types, Nusbaum and Marder, 1989; Coleman et al., 1992; Follmann et al., 2017) are located upstream in the bilateral symmetric commissural ganglia (CoG, Figure 1A). Four commissural projection neurons have been characterized in detail: modulatory commissural neurons 1, 5, and 7 (MCN1, MCN5, MCN7) and commissural projection neuron 2 (CPN2) (Nusbaum and Beenhakker, 2002). They are activated by several different sensory neurons and pathways that convey food availability (Beenhakker et al., 2004; Hedrich et al., 2009). The mechanosensory ventral cardiac neurons (VCN, ∼40 bilateral copies), for example, respond to increased mechanical pressure in the cardiac gutter region of the foregut. VCN activation leads to a long-lasting excitation of MCN1 and CPN2 (Figure 1B), which is necessary and sufficient to start a particular version of the gastric mill rhythm, the VCN gastric mill rhythm (Beenhakker and Nusbaum, 2004). Powell et al. (2021) recently demonstrated that the VCN gastric mill rhythm shows considerable robustness against temperature changes when both the gastric mill CPG and the extrinsic projection neurons in upstream ganglia (commissural ganglia and esophageal ganglion, Figure 1A) are heated. Our previous experiments show that another version of the gastric mill rhythm – elicited by direct stimulation of MCN1 – is profoundly temperature-sensitive when only the gastric mill CPG in the STG is heated, but MCN1 activity remains unaffected by temperature (Städele et al., 2015). In contrast, when MCN1’s activity increases, the gastric mill rhythm stays functional over a broader temperature range. Whether such extrinsic neuromodulation is necessary to achieve temperature robustness for other versions of the gastric mill rhythm, including the mechanosensory elicited VCN rhythm, remains unclear.

To address this, we independently modified the temperature of the STG circuits and the projection neurons in the upstream esophageal (OG) and commissural ganglia (Figure 1A). For starters, we measured temperature responses of spontaneously active gastric mill rhythms. Figure 1C shows the result of such an experiment where both the STG and the upstream ganglia were kept at 10°C (top traces). When we selectively increased the temperature of the STG but kept upstream ganglia at 10°C, we found that the rhythm continued at 12°C but ceased at 14.5°C. Increasing the STG temperature further to 16°C did not reestablish the rhythm. However, when we lowered the STG temperature back to 12°C, the rhythm reappeared and continued at 10°C. We observed similar behaviors in all preparations tested (*N* = 5), with spontaneous gastric mill rhythms crashing at temperatures below 19°C. Together, our findings suggest that the broad temperature robustness of spontaneous gastric mill rhythms depends at least partly on heating the upstream ganglia and thus on factors extrinsic to the CPG network in the STG. This conclusion is consistent with our previous results showing that extrinsic peptide modulation bestows temperature robustness of the MCN1 gastric mill rhythm (Städele et al., 2015). Given that spontaneous gastric mill rhythms rarely are generated by MCN1 alone, our data indicate that extrinsic modulation is also a critical factor in sustaining other types of gastric mill rhythms.

### Ventral Cardiac Neurons Gastric Mill Rhythms Are Not Temperature Robust Without Neuromodulatory Compensation

To further investigate the impact of extrinsic modulatory factors, we bilaterally stimulated the VCNs via the *dpon* nerve through which the VCN axons project to the commissural ganglia (Figures 1A,B). VCN stimulation activates the projection neurons MCN1 and CPN2, which in turn drive the gastric mill rhythm (Beenhakker and Nusbaum, 2004; Blitz et al., 2004). We used identical stimulus protocols as Powell et al. (2021). Consistent with previous data, bilateral *dpon* stimulation elicited long-lasting (>15 min) gastric mill rhythms at control temperature (9–10°C) in each preparation tested. We then selectively altered STG temperatures (7–22°C, in 3°C increments), stimulated again, and tested whether gastric mill rhythms could be elicited. If a gastric mill rhythm occurred, we measured characteristics such as cycle period, rhythm duration, burst durations of the neurons, and their phase relationship. It is well-established that the rhythm structure of VCN gastric mill rhythms varies substantially directly after the end of the *dpon* stimulation (Beenhakker et al., 2004). We thus analyzed 5 min after the end of the *dpon* stimulation, when the rhythm had stabilized. Unless otherwise stated, we kept the temperature of upstream extrinsic projection neurons constant between 9 and 10°C (control temperature) to separate temperature effects on the gastric mill CPG from those extrinsic to it. Thus, the projection neurons activated by *dpon* stimulation and driving the VCN gastric mill rhythm (MCN1 and CPN2) remained at control temperature, while the gastric mill CPG network in the STG was exposed to changing temperatures. We did not test temperature effects on VCN rhythms elicited with unilateral *dpon* stimulation. Typically, unilateral *dpon* stimulation elicits shorter and weaker rhythms because mainly the projection neurons of only one of the two commissural ganglia are participating in the rhythm. Temperature effects on the STG gastric mill circuits are thus likely to be even more pronounced with unilateral *dpon* stimulation.

**FIGURE 2:**
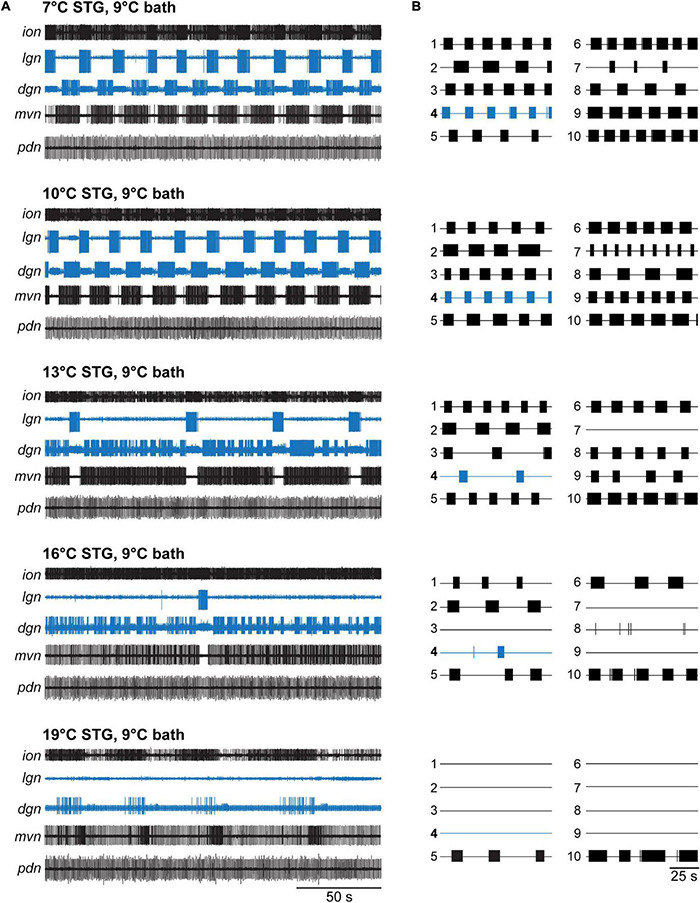
VCN gastric mill rhythms crash when only the STG is heated. **(A)** Example extracellular nerve recordings of both the gastric mill (*lgn*, *dgn*) and pyloric (*pdn*) rhythms at different STG temperatures. Recordings of the gastric mill neurons LG (on the *lgn*) and DG (on *dgn*) are highlighted in blue. The *pdn* recording shows the activity of the PD neurons as a representation of the pyloric rhythm. For completeness, the activity of the inferior cardiac (IC) and ventricular dilator (VD) neurons (both gastro-pyloric neurons) on the *mvn* is plotted as well. Note, the gastric mill (GM) neurons, which participate in the VCN rhythm, are absent from the *mvn* because the recording was taken peripherally, after all GM axons had left this nerve. For each temperature, a gastric mill rhythm was elicited with *dpon* stimulation. Bath temperature was kept constant at 9°C while STG temperature was increased. At elevated STG temperatures (16 and 19°C), *dpon* stimulation failed to elicit a gastric mill rhythm. **(B)** Summary of all experiments (*N* = 10 preparations). Each numbered trace represents the spike activity of LG over 100 s for one experiment, recorded 300 s after the end of the *dpon* stimulation. The blue trace corresponds to the example shown in **(A)**.

Figure 2A shows original recordings at STG temperatures of 7–19°C in 3°C steps. At 13°C, the gastric mill rhythm became irregular and was essentially absent at higher temperatures. Specifically, the core CPG neuron LG showed regular rhythmicity and burst activity at 7 and 10°C. LG is part of the half-center circuit of the gastric mill CPG and required for gastric mill activity. Behaviorally, it controls the protraction of the two lateral teeth in the gastric mill of the foregut. At 13°C, there were long pauses between LG bursts, and the cycle period started to vary. At 16°C, only a single LG burst was present, and no LG bursts occurred at 19°C (= crash). Figure 2A also shows the dorsal gastric neuron DG (see Figure 1B for connectivity). DG is a gastric mill follower neuron whose activity roughly antagonizes that of the LG neuron (Coleman et al., 1995). Behaviorally, DG is the retractor of the single medial tooth, and thus a functional antagonist to LG. DG showed regular rhythmic activity at 7 and 10°C, became arrhythmic with vastly varying burst durations at 13 and 16°C, and recovered a slow rhythmic activity with varying burst durations at 19°C.

In contrast to the gastric mill rhythm, the pyloric rhythm continued to produce regular activity across all temperatures tested, consistent with previously published data (Tang et al., 2010; Soofi et al., 2014). Figure 2A shows this with a recording of the pyloric dilator neurons (PDs) on the *pdn*. The PDs are part of the pyloric pacemaker ensemble, together with the anterior burster (AB) and lateral posterior gastric (LPG) neurons (Figure 1B). The PDs are traditionally used as a convenient measure for pyloric activity.

**FIGURE 3:**
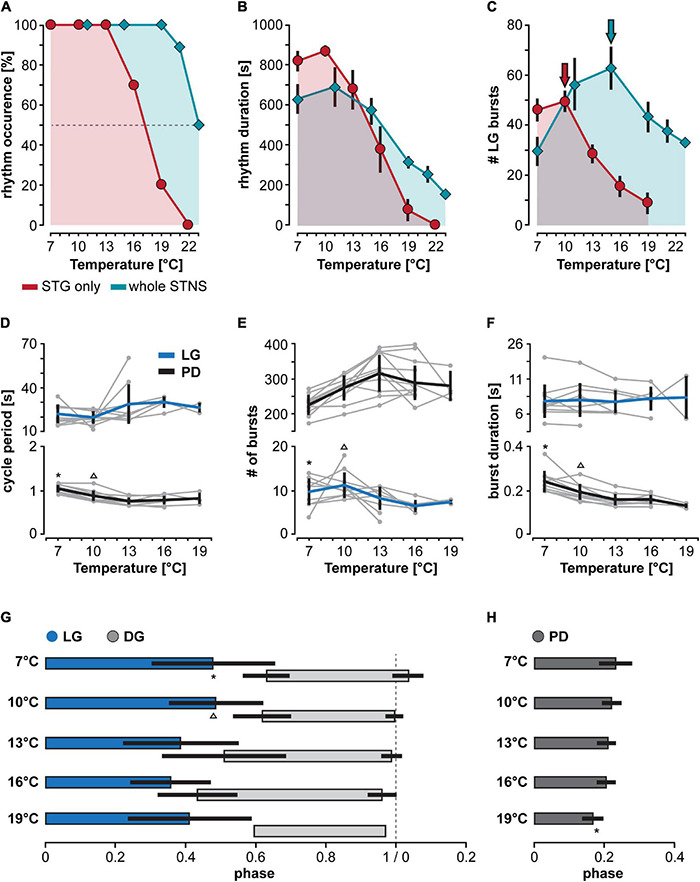
Heating extrinsic input fibers improves temperature resistance of CPGs in the STG. **(A–C)** Comparison of gastric mill rhythm parameters for two different heating conditions. Red traces: STG temperature changed while CoGs were kept at 9°C. Teal traces: STG and CoG temperatures were changed together (reanalyzed data from Powell et al., 2021). Dots are representing mean values ± SEM of 10 preparations. For better comparison, areas underneath the curves were colored. **(A)** Number of gastric mill rhythm occurrences at different temperatures. **(B)** Gastric mill rhythm duration, and **(C)** LG spike number at different temperatures. **(D–F)** Responses of pyloric and gastric mill rhythms when only the STG is heated. Cycle periods of LG (blue) and pyloric dilator (PD, black) are shown across temperature. Thick lines represent the mean ± SEM for 10 preparations tested. Individual experiments are depicted in gray. Dots represents mean values of data collected over 200 s, recorded 300 s after the end of the *dpon* stimulation. The gastric mill rhythm crashed at varying temperatures, visible by the decline in the number of gray dots for LG at higher temperatures. **(D)** LG and PD cycle period across temperature. Statistics: LG cycle period: not significant different, One Way RM ANOVA, *N* = 10 (7–10°C), *N* = 9 (13°C), *N* = 5 (16°C), *N* = 2 (19°C), *F*(4,22) = 2.436, *p* = 0.078. PD cycle period: One Way RM ANOVA, *N* = 10 (7–16°C), *N* = 3 (19°C), *F*(4,29) = 42.651, *p* < 0.001, significantly different for 7°C vs. all other temperatures (indicated with *), and 10°C vs. all others (indicated with Δ), *p* < 0.05, Student–Newman–Keuls *post hoc* test. **(E)** Number of LG and PD bursts across temperature. LG bursts: not significant different, One Way RM ANOVA, *N* = 10 (7–10°C), *N* = 9 (13°C), *N* = 5 (16°C), *N* = 2 (19°C), *F*(4,22) = 2.582, *p* = 0.065. PD bursts: One Way RM ANOVA, *N* = 10 (7–16°C), *N* = 3 (19°C), *F*(4,29) = 11.851, *p* < 0.001, significantly different for 7°C vs. 13° and 16°C (*). 10°C was also significantly different from 13°C (Δ), *p* < 0.05, Student–Newman–Keuls *post hoc* test. **(F)** LG and PD burst duration across temperature. LG burst duration: no significant change, One Way RM ANOVA, *N* = 10 (7–10°C), *N* = 9 (13°C), *N* = 5 (16°C), *N* = 2 (19°C), *F*(4,22) = 1.067, *p* = 0.396. PD burst duration: One Way RM ANOVA, *N* = 10 (7–16°C), *N* = 3 (19°C), *F*(4,29) = 22.752, *p* < 0.001, significant differences for 7°C (*) and 10°C (Δ) vs. all higher temperatures, *p* < 0.05, Student–Newman–Keuls *post hoc* test. **(G)** Phase relationship and duty cycle of LG (blue), DG (light gray), and PD **(H)** across various STG temperatures. LG duty cycle (= end of its activity phase): One Way RM ANOVA, *N* = 10 (7–10°C), *N* = 9 (13°C), *N* = 5 (16°C), *N* = 2 (19°C), *F*(4,22) = 10.242, *p* < 0.001, significant differences for 7°C (*) and 10°C (Δ) vs. all higher temperatures, *p* < 0.05, Student–Newman–Keuls *post hoc* test. At 19°C, DG was only active in one animal. DG phase onset: significant effect of temperature (*p* = 0.018), but no significant difference between any two conditions. One way ANOVA, *N* = 8 (7°C), *N* = 10 (10°C), *N* = 9 (13°C), *N* = 5 (16°C), *N* = 1 (19°C), *F*(3,28) = 3.938, *p* = 0.018, *p* > 0.05 for all paired comparisons, Student–Newman–Keuls *post hoc* test. DG phase end: One way ANOVA, *N* = 8 (7°C), *N* = 10 (10°C), *N* = 9 (13°C), *N* = 5 (16°C), *N* = 1 (19°C), *F*(3,28) = 5.395, *p* = 0.005, significant differences between 7 and 16°C (*), *p* < 0.05, Student–Newman–Keuls *post hoc* test. DG duty cycle: no significant effect of temperature, One way ANOVA, *N* = 8 (7°C), *N* = 10 (10°C), *N* = 9 (13°C), *N* = 5 (16°C), *N* = 1 (19°C), *F*(3,28) = 2.786, *p* = 0.059. PD: One Way RM ANOVA, *N* = 10 (7–16°C), *N* = 3 (19°C), *F*(4,29) = 6.019, *p* = 0.001, significant differences for 19°C vs. all others (*), *p* < 0.05, Student–Newman–Keuls *post hoc* test.

Our data were consistent across preparations tested (Figure 2B), although the lowest temperature at which stimulation failed to elicit a lasting rhythm varied (13°C: *N* = 1/out of 10 preparations crashed, 16°C: *N* = 3/10, 19°C: *N* = 9/10, 22°C: *N* = 10/10). In all temperature conditions, VCN stimulation elicited the typical responses during stimulation (Beenhakker et al., 2004). In some cases, when the rhythms crashed, a few cycles of gastric mill activity were observed immediately after the end of the stimulation. However, this activity was short-lived and never continued. Figure 3A (red) shows the percentage of rhythms present 5 min after *dpon* stimulation for the various temperatures tested. For analysis, we scored rhythms as present when at least 3 LG bursts occurred within 200 s, no matter how irregular their occurrence was.

The crash of the gastric mill rhythm at relatively low temperatures contrasts with findings from Powell et al. (2021) where the CoGs were heated to the same temperature as the STG. For comparison, we thus reanalyzed publicly available data (Powell et al., 2021) and plotted them next to ours (Figure 3A, teal). The difference is quite striking in that all rhythms where the bath (and thus also the CoGs) was kept at control temperatures crashed at lower or equal temperatures than the rhythms where STG and CoG temperature were the same. This difference was also reflected in rhythms generally ending earlier after the *dpon* stimulation when only the STG was heated (Figure 3B), with correspondingly fewer total LG bursts (Figure 3C). We noted that independent of the heating condition (entire preparation vs. STG only), the total number of LG bursts generated after *dpon* stimulation reached a peak before decreasing at higher temperature. When only the STG was heated, the peak occurred at 10°C in comparison to 16°C when the entire preparation was heated (Figure 3C, arrows).

### Extrinsic Factors Contribute to the Temperature Responses of Pyloric and Gastric Mill Rhythms

To further characterize the temperature response of the gastric mill rhythm, we analyzed the gastric mill cycle period, the number of bursts per time, LG burst duration, and LG duty cycle (over 200 s). We noted a pronounced difference to data from Powell et al. (2021). In our experiments, where only the STG was heated, the gastric mill period did not decrease dramatically with temperature (Figure 3D). Instead, the cycle period tended to increase with temperatures over 10°C, albeit not significantly. Similarly, the number of LG bursts per time remained unchanged (Figure 3E). LG burst duration was also constant despite the increasing temperature (Figure 3F). We found, however, that because of the trend toward increasing cycle periods and constant burst durations, LG’s duty cycle in the rhythm decreased at 13°C and above (Figure 3G). The decrease in LG duty cycle was accompanied by an increase in the duty cycle of the DG neuron (Figure 3G), LG’s functional antagonist. With respect to the behavioral output of the rhythm, this shortens the protraction phase and extends the retraction phase of the neurons that control tooth movements in the crab foregut. Together, our data demonstrate that dissociating temperature effects on the CPG from those of upstream ganglia substantially lowered the robustness of the gastric mill rhythm against temperature perturbation, suggesting that factors extrinsic to the CPG provide a functionally relevant component to temperature compensation.

We further noted that the variability of the rhythm did not increase significantly when approaching the crash temperature. The original recordings in Figure 2 already show this trend in that there was either regular rhythmic burst activity or no burst activity at all. We also did not find quantitative differences in rhythm variability when we compared the coefficient of variation of the gastric mill cycle period at 10°C and at the highest temperature at which a rhythm could still be elicited (10°C: 0.11 ± 0.07, pre-crash temperature: 0.17 ± 0.13; paired *t*-test, *N* = 10, *P* = 0.134).

While the pyloric rhythm continued to function in all of our experiments, even at temperatures where the gastric mill rhythm had crashed, we noted more differences to previous experiments in which CoGs and STG were heated together (Powell et al., 2021). Most obviously, although there was a significant decrease in the pyloric cycle period at temperatures of 13°C and above (Figure 3D), this change seemed modest when compared to Powell et al. (2021). To determine the pyloric cycle period, we again used the bursts of the pyloric dilator (PD) neurons on the *pdn* and measured the time between their onsets. In agreement with the small effect on the cycle period, there was no consistent change in the total number of PD bursts (Figure 3E). This suggests that some aspects of the speeding up of the pyloric rhythm that is observed in intact animals (Soofi et al., 2014) and during heating of the entire nervous system (Tang et al., 2010, 2012; Haddad and Marder, 2018; Powell et al., 2021) are likely to be mediated by extrinsic factors.

We also found similarities to previously published data. For example, like previously described for varying temperatures both *in vitro* and *in vivo* (Tang et al., 2010; Soofi et al., 2014), the duty cycle of the pyloric PD neurons (their burst duration relative to the cycle period) remained mainly constant throughout the temperature range tested. Accordingly, PD burst duration (Figure 3F) mirrored the pyloric cycle period in that it shortened significantly at temperatures of 13°C and above, which together lead to a primarily constant duty cycle. Only when the temperature reached 19°C, there was a significant shortening of the duty cycle (Figure 3H).

Taken together, our data demonstrate that factors extrinsic to the STG, and thus to the pyloric and gastric mill CPGs, contribute to the temperature responses of both rhythms. For the gastric mill rhythm, neurons residing outside the STG appear to provide a significant component to the substantial temperature robustness of the rhythm.

### Gastric Mill Rhythm Rescue Is Accompanied by Temperature-Induced Increase in Modulatory Projection Neuron Activity

In previous studies (Städele et al., 2015; DeMaegd and Stein, 2021), we have shown that extrinsic modulation enables the temperature robustness of a specific version of the gastric mill rhythm, elicited by the paired descending modulatory projection neuron MCN1. Specifically, the peptide co-transmitter of MCN1, CabTRP Ia, reduces shunting in LG to counterbalance an excessive increase in LG leak currents. CabTRP Ia is released increasingly with higher MCN1 firing frequencies (Stein et al., 2007). The increased MCN1 firing frequency strengthens the robustness of the gastric mill rhythm against temperature perturbations (Städele et al., 2015).

MCN1 also contributes to the VCN version of the gastric mill rhythm investigated here. VCN stimulation co-activates MCN1 with a second projection neuron, CPN2, and together they drive the rhythm. MCN1 releases only one peptide that affects LG (CabTRP Ia) (Nusbaum and Beenhakker, 2002; Stein et al., 2007). While CPN2’s neurotransmitters are unknown, most of its actions in the STG are mediated through electrical synapses. CPN2’s effects on LG are weak compared to MCN1 (Stein et al., 2007) but lead to the recruitment of additional gastric mill neurons into the rhythm (Beenhakker and Nusbaum, 2004). None of these additional neurons are necessary for rhythm generation, suggesting that MCN1 is the main contributor to LG excitation and rhythm generation. We thus hypothesized that the temperature robustness of the VCN gastric mill rhythm is enabled, at least in part, by extrinsic peptide modulation from MCN1.

**FIGURE 4:**
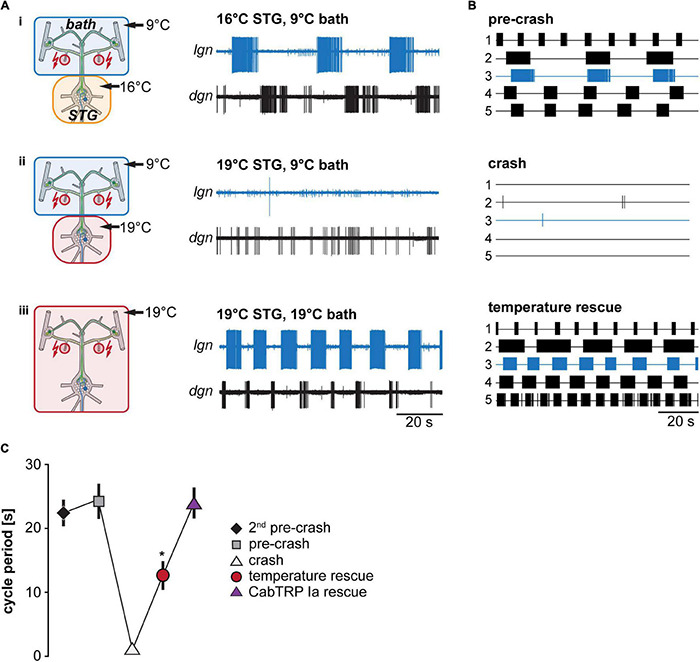
Matching STG and CoG temperature prevents gastric mill rhythm crash. **(A)** Schematic representation of the temperature conditions (left) and the corresponding activity of LG and DG in one preparation (right). **(B)** Summary of all experiments (*N* = 5). Each trace represents the spike activity of LG over 100 s for an individual experiment, recorded 300 s after the end of the *dpon* stimulation. Blue traces correspond to data shown in **(A)**. Crash temperatures varied between experiments. Conditions are labeled as pre-crash, crash, and temperature rescue. **(C)** Comparison of LG cycle period for the specified conditions. Shown are mean values ± SEM of all preparations (*N* = 10). For completeness, LG cycle period during bath application of CabTRP Ia (Figure 6) was added to this plot (*N* = 9). LG cycle period was significantly decreased during temperature rescue compared to the 2nd pre-crash, pre-crash, and CabTRP Ia rescue (indicated with *). One Way ANOVA, *F*(3,30) = 55.972, *p* = 0.046, *p* < 0.05 for paired comparisons, Student–Newman–Keuls *post hoc* test.

We tested whether temperature-induced up-regulation in projection neuron firing frequency is sufficient to prevent the termination of the VCN gastric mill rhythm at elevated temperatures (Figure 4). Like in our previous experiments, we stimulated the *dpon* to elicit a VCN gastric mill rhythm while we heated the STG to different temperatures. We then determined the crash temperature, i.e., the temperature at which stimulation failed to elicit a gastric mill rhythm. The ganglia extrinsic to the STG were then also heated to the crash temperature. The *dpon* was stimulated again with both, the STG and the CoGs, at crash temperature. Figure 4A shows a representative recording of LG at 16°C (trace i), where the rhythm could still be elicited, and then at a crash temperature of 19°C (ii), where the rhythm failed when only the STG was heated. After additionally heating the CoGs to 19°C (iii), however, the gastric mill rhythm could be recovered with *dpon* stimulation. In all preparations tested (*N* = 5, Figure 4B), heating of the STG and CoGs together led to a rescue of gastric mill activity at crash temperature. We further noted that the rhythm sped up during this rescue, with a significantly shorter cycle period than at the last temperature before the crash (pre-crash, Figure 4C). This result is consistent with the observation of Powell et al. (2021) that increasing temperatures caused faster rhythms when STG and CoGs were heated together. Our data also suggest that a heat-activated factor extrinsic to the STG is sufficient to enable temperature robustness of the gastric mill rhythm.

**FIGURE 5:**
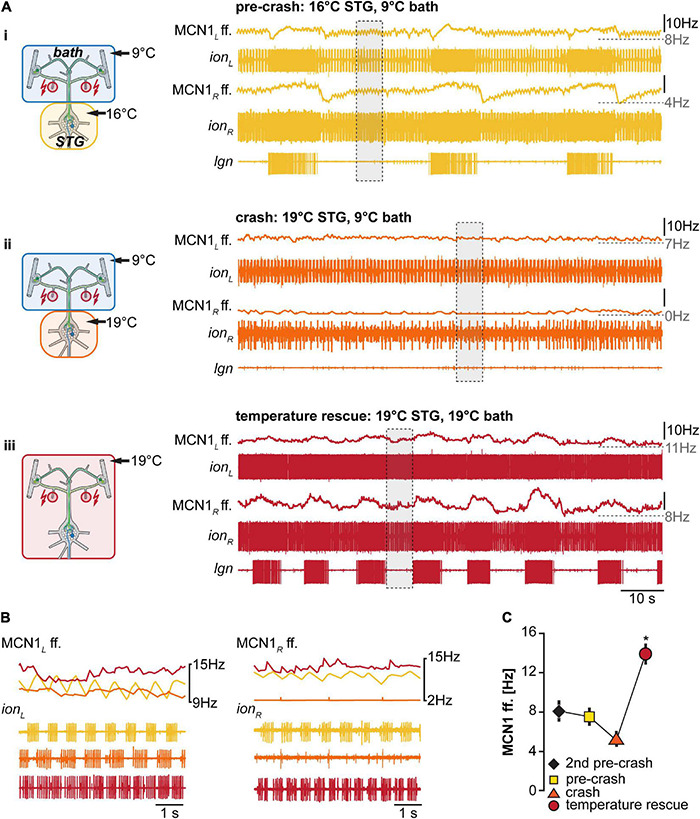
Matching STG and CoG temperature increases MCN1 firing frequency. **(A)** Schematic representation of the temperature conditions (left) and the corresponding activity of MCN1 of one preparation (right). Firing frequencies for each of the two MCN1 neurons are shown. MCN1_L_, left MCN1; ion_L_, left ion; MCN1_R_, right MCN1; ion_R_, right ion. Traces above each *ion* recording show the mean MCN1 firing rate (sliding window of 1 s). The bottom recording shows the *lgn*, and the LG neuron and gastric mill rhythm. **(B)** Top: Comparison of MCN1 mean firing frequency for both MCN1 neurons (MCN1_L_ and MCN1_R_). Bottom: expanded view of the *ion* recordings depicted in **(A)** with gray boxes. Colors represent the temperature conditions shown in **(A)**. **(C)** Comparison of mean MCN1 firing frequency for all experiments (*N* = 4 for temperature rescue, *N* = 8 all other conditions). MCN1_L_ and MCN1_R_ frequencies were averaged. The MCN1 firing frequency in the temperature rescue condition was significantly increased in comparison to all other conditions (indicated with *). One Way ANOVA, *F*(3,24) = 5.102, *p* < 0.001, *p* < 0.05 for all paired comparisons, Student–Newman–Keuls *post hoc* test.

Our previous work had indicated that the activity of the CoG projection neurons MCN1 increases with temperature (Städele et al., 2015), albeit in different experimental conditions. We hypothesized that MCN1 and its co-transmitters contribute to the temperature robustness of the VCN gastric mill rhythm and predicted that its activity increases when both the STG and CoGs are warmed to the same temperature. Conversely, we also predicted that MCN1 activity remains unchanged when only the STG is heated, ultimately leading to a crash of the gastric mill rhythm. To test these predictions, we investigated MCN1’s firing frequency after *dpon* stimulation in the different STG temperature conditions (STG heated vs. entire preparation heated). Figure 5A shows original recordings of the two MCN1 neurons on the left and right inferior esophageal nerves (*ion*) and the corresponding MCN1 firing frequencies for three different temperature conditions. LG activity was monitored extracellularly on the *lgn*. At pre-crash temperature (STG 16°C, CoGs 9°C, Figure 5Ai), VCN stimulation elicited a regular, slow gastric mill rhythm. MCN1’s firing frequency varied rhythmically, with the highest frequencies coinciding with the LG burst onset and lowest frequencies immediately following the LG burst end. This progression of firing frequencies is reminiscent of previously published data (Beenhakker and Nusbaum, 2004) and partly depends on feedback from the gastric mill CPG to the commissural projection neurons (Blitz, 2017).

Figure 5Aii shows MCN1 firing frequencies after the rhythm had crashed (STG 19°C, CoGs 9°C). Note that MCN1 firing frequencies were similar to the lowest frequencies of the pre-crash temperature. However, LG bursts were absent, indicating that MCN1 could not recruit LG, so the gastric mill rhythm crashed. In fact, LG was silent, stopping CPG oscillation entirely. As a consequence of the missing CPG feedback to the commissural projection neurons, rhythmic increases in MCN1 firing frequency were also absent in this condition, further lowering the chances of recruiting LG. Bringing the CoGs to the same temperature as the STG circuits (both at 19°C) caused an increase in MCN1 firing frequencies and restored the rhythm (Figure 5Aiii). Figure 5B compares MCN1 firing frequencies for the areas highlighted with gray boxes in Figure 5A, separated for the left and right MCN1. Examples were taken from the LG interburst period, i.e., when MCN1 activities were lowest during rhythms. The comparison shows that MCN1 activity was lowest at the crash temperature, and it was highest in the temperature rescue condition. We found this to be true for all preparations tested (*N* = 4). Figure 5C shows the averaged summary of all experiments, including two temperatures before the rhythm crashed. There was no apparent effect on MCN1 firing frequency at the two temperatures before the crash. The lowest MCN1 activity was consistently observed at crash temperature, in accordance with the lack of gastric mill rhythm and STG circuit feedback to the CoGs. Importantly, when the CoGs were heated to the crash temperature (temperature rescue), MCN1 activity increased significantly, and the rhythm was restored. These data are consistent with the idea that increased MCN1 activity rescued the gastric mill rhythm.

### Neuromodulator Application Rescues the Gastric Mill Rhythm

**FIGURE 6:**
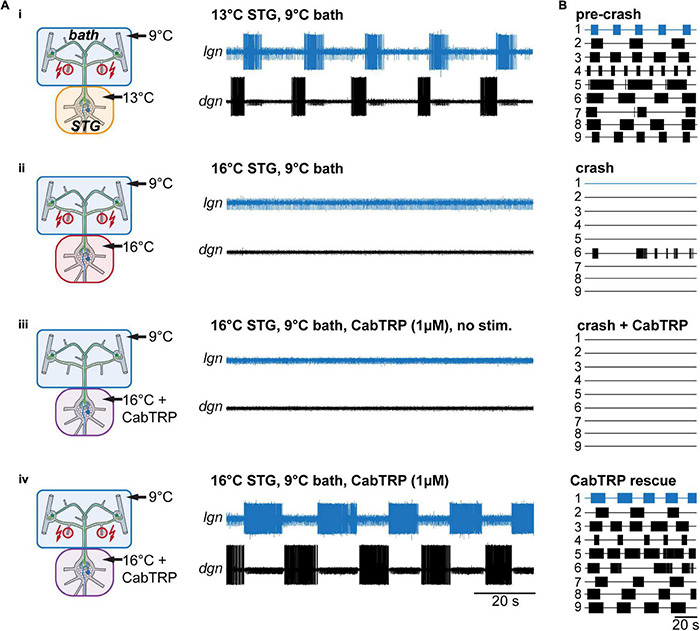
Rhythms can be recovered by bath application of MCN1’s peptide co-transmitter CabTRP Ia. **(A)** Schematic representation of the temperature conditions (left) and the corresponding activity of LG and DG of one preparation (right). **(B)** Summary of all experiments (*N* = 9). Each numbered trace represents the spike activity of LG over 100 s for one experiment, recorded 300 s after the end of the *dpon* stimulation. The blue traces correspond to the experiment shown in **(B)**.

MCN1’s neuropeptide co-transmitter CabTRP Ia has previously been indicated to provide temperature-robustness to LG’s activity (Städele et al., 2015). These experiments, however, had been done for the MCN1-only gastric mill rhythm. We suspected that CabTRP Ia might have a similar effect on the VCN gastric mill rhythm. To test this, we again elicited gastric mill rhythms by stimulating the *dpons* (Figure 6Ai), increased the STG temperature until no rhythm could be elicited anymore (Figure 6Aii), and then bath applied CabTRP Ia (1 μM) selectively to the STG. The CoGs were always kept at control temperature (9°C) and in regular saline. Applying CabTRP Ia by itself did not alter LG activity, i.e., no gastric mill rhythm was elicited by simply adding CabTRP Ia (Figure 6Aiii). However, stimulating the *dpon* in CabTRP Ia restored the gastric mill rhythm at crash temperature (Figure 6Aiv), resembling the situation in the MCN1-only rhythm (Städele et al., 2015) and the temperature rescue of the VCN rhythm (Figure 5Aiii). In all preparations tested (*N* = 7), CabTRP Ia application was sufficient to restore the VNC rhythm at elevated temperatures (Figure 6B). We noted however that unlike in the temperature rescue experiments in which the CoGs and STG were heated together (Figures 5A,B), rhythms in CabTRP Ia showed no significant change in cycle period to the pre-crash rhythms in saline (Figure 4C). This suggested that the continuous presence of MCN1’s co-transmitter is insufficient to establish the temperature-induced increase in gastric mill rhythm speed when the whole nervous system is heated. Instead, speed control of the rhythm may be reliant on the timing cues of CabTRP Ia release by MCN1.

In summary, our data show that MCN1’s co-transmitter CabTRP Ia can stabilize the VCN gastric mill rhythm over a broad temperature range. Our data further suggest that increased MCN1 activity and CabTRP Ia release are critical components of the temperature robustness of gastric mill rhythms that involve MCN1.

### Integer Coupling Is Determined by Stomatogastric Ganglion Circuit Architecture and Does Not Depend on Extrinsic Neuromodulation

**FIGURE 7:**
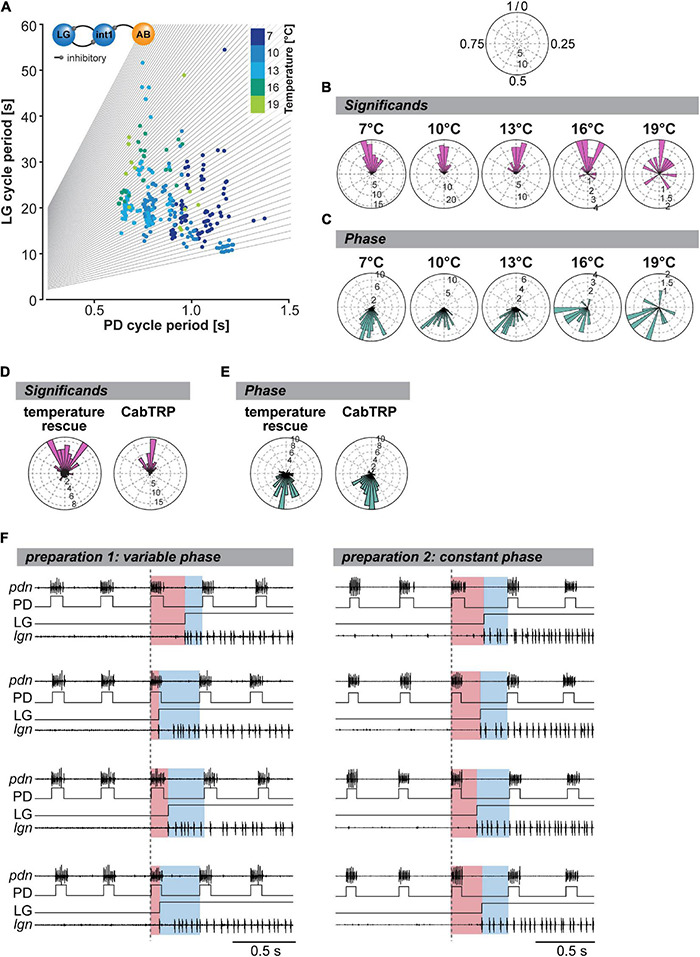
Integer coupling is always present when gastric mill and pyloric rhythms are active but weakens at elevated temperature. **(A)** LG cycle period as a function of mean PD period (= mean of all PD bursts within one LG cycle period). Gray lines represent the integer slopes and data points with integer coupling lie along these lines. *N* = 10 preparations. Inset: The synaptic connectivity between the pyloric pacemaker AB and the gastric mill CPG neurons (Interneuron 1 and LG) underlies integer coupling between pyloric and gastric mill rhythms. **(B)** Significant for all tested preparations (*N* = 10) shown as rose plots for various STG temperatures. Integer coupled data points will coincide at the top of the diagram (close to 1/0, see schematic at the top). Vector strengths are indicated by the radius of the plot. Analyzed were 200 s of recordings, 300 s after the last *dpon* stim. Rayleigh *z* Test: 7°C: Rayleigh *z*_65.6_, *p* < 0.001, 10°C: Rayleigh *z*_83.8_, *p* < 0.001, 13°C: Rayleigh z_49.7_, *p* < 0.001, 16°C: Rayleigh *z*_12.6_, *p* < 0.001, 19°C: Rayleigh z_3.0_, *p* = 0.045. **(C)** Analysis of phase coupling of the LG and PD burst onset at various temperatures (*N* = 10 preparations). Rayleigh *z* Test: 7°C: Rayleigh *z*_64.2_, *p* < 0.001, 10°C: Rayleigh *z*_77.3_, *p* < 0.001, 13°C: Rayleigh *z*_46.3_, p < 0.001, 16°C: Rayleigh *z*_12.8_, *p* < 0.001, 19°C: Rayleigh *z*_3.5_, *p* = 0.027. **(D)** Significant of all gastric mill cycles and animals for the temperature rescue (*N* = 10) and CabTRP Ia (1 μM, *N* = 9) experiments. Rayleigh *z* Test: temperature rescue: Rayleigh *z*_24.3_, *p* < 0.001; CabTRP Ia rescue: Raleigh *z*_47.2_, *p* < 0.001. **(E)** Phase coupling of the LG and PD burst onsets for the temperature rescue and CabTRP Ia (1 μM) experiments. Rayleigh *z* Test: temperature rescue: Rayleigh *z*_38.5_, *p* < 0.001; CabTRP Ia rescue: Rayleigh *z*_48.9_, *p* < 0.001. **(F)** Example recordings from two preparations during temperature rescue. The LG burst is shown with a recording of the *lgn* and schematized LG bursts (LG). Four individual LG burst onsets are displayed (top to bottom) for each preparation. Pyloric cycles are shown by recordings of the PD neurons (*pdn*) and schematized PD bursts (PD). The dashed vertical line indicates the beginning of the last PD burst before the LG burst onset. The time delay between PD onset and LG onset is highlighted in red. The remainder of the pyloric cycle is marked in blue. A change in the ratio of red and blue illustrates a shift in the phase relationship between PD and LG onset. The phase relationship varied for the preparation shown on the left but not for the one on the right.

Powell et al. (2021) have shown that integer coupling between the pyloric rhythm and the VCN gastric mill rhythm is robust over a broad temperature range (up to 25°C). While the details for the VCN-elicited gastric mill rhythm have not been investigated, studies on the MCN1-only rhythm have shown that the integer coupling is mainly mediated by the pyloric pacemakers’ synaptic inhibition to Interneuron 1 (Int1), the half-center antagonist of LG in the gastric mill CPG. Rhythmic pyloric activity of the AB pacemaker neuron inhibits Int1 (Figures 1B, 7A, inset), which results in disinhibition of LG, enabling LG to burst in synchrony with the pyloric pacemakers (Nadim et al., 1998; Bartos et al., 1999). LG bursts are thus initiated shortly after Int1 is inhibited by the pyloric pacemakers. As a consequence of the pyloric-timed disinhibition of LG, each gastric mill cycle is comprised of an integer multiple of pyloric cycles. There are also indications that integer coupling may be present *in vivo* (Hedrich et al., 2011; Yarger and Stein, 2015).

While not explicitly mentioned, the work of Powell et al. (2021) suggests that LG disinhibition, a CPG-intrinsic connection, is also the primary mechanism determining the timing of the VCN rhythm, no matter the temperature. Consequently, integer coupling should be independent of neuromodulatory input and maintained across temperatures as long as both rhythms are active. To test this, we again changed the temperature of only the STG circuits and measured the coupling strength of the two rhythms by analyzing the relationship between gastric mill (LG) and pyloric (PD) cycle period (Figure 7A). As expected for integer coupling, most of our data tended to lie along lines with integer slope (gray lines). Only a few data points were between slopes, demonstrating that integer coupling was independent of STG temperature. Our data thus support the hypothesis that the mechanisms establishing integer coupling are intrinsic to the CPG circuits within the STG. Integer coupling does not strongly rely on extrinsic factors to be maintained when the temperature changes. However, we noted that in our data set there were fewer data points near the axes’ origins than when STG and CoGs were heated together (comparison with Figure 5B, Powell et al., 2021). In our experiments where only the STG was heated, the gastric mill rhythm slowed down at higher temperatures while the pyloric rhythm sped up. In contrast, when STG and CoGs were heated together (Powell et al., 2021), both rhythms sped up, resulting in more cycles with short cycle periods and thus more data near the axes’ origins.

To further quantify integer coupling, we calculated the significant according to Powell et al. (2021). The significant provides a convenient number to assess integer coupling by providing a single number for each gastric mill cycle. Integer coupling exists when the significant value is either close to zero or close to one. To compare the effect of temperature on integer coupling, we plotted the significant in a rose diagram (Figure 7B). This representation is meaningful because rhythmic patterns are inherently comprised of repetitive circular patterns, and the end of one cycle represents the beginning of the next. We defined the beginning of the pyloric cycle as 0 and the end as 1. In this view, significant values near 0 and 1 coincide at the top of the diagram, providing a continuous measure for integer coupling. We plotted the significant of all gastric mill cycles and experiments separately for each temperature condition and measured the resulting vector strength. We found that, as long as the gastric mill rhythm was active, the resulting vector was significantly different from an equal distribution, supporting the Null hypothesis that most LG bursts occurred after an integer number of pyloric cycles (Figure 7B). Thus, the two rhythms were integer coupled at all temperatures, even when only the STG was heated. However, we noted that the *z* values of our Rayleigh analysis diminished with higher temperatures, suggesting a wider distribution of significant and thus a weakened integer coupling. The weakened integer coupling was also evident from the wider spread of data points in the rose plots at 16 and 19°C (Figure 7B).

If integer coupling between the pyloric and gastric mill rhythms depends on LG disinhibition when the pyloric pacemaker neuron AB inhibits Int1 (Bartos et al., 1999), the LG burst should start at a fixed phase of the pyloric cycle. We tested whether this was the case by plotting the phase of the LG burst onset within the pyloric cycle, again using rose plots (Figure 7C). A pyloric cycle was defined from the start of the PD neuron burst to the beginning of the subsequent PD burst (see section “Materials and Methods”). The cycle period was calculated using the average of the two cycles preceding the LG burst. The onset phase of the LG burst was then calculated as the time delay with which the LG burst began after the start of the PD burst, divided by the pyloric cycle period. As expected for integer coupling, the observed phases clustered within a small range at low temperatures. Like for the significant, the averaged resulting phase vector was statistically different from a random distribution at all temperatures (Figure 7C). Thus, both measures, significant and phase, demonstrated that integer coupling was present. However, the range of observed phases increased at 16 and 19°C when compared to colder temperatures. While still statistically different from a random distribution and thus integer coupled, the coupling appeared to weaken at 16°C and above.

Per definition, no integer coupling can exist between the pyloric and gastric mill rhythms at crash temperature when the gastric mill rhythm stops. However, since rhythms continue to function when STG and CoGs are heated together, or CabTRP Ia is present, we asked whether integer coupling is maintained when the rhythm is rescued. For analysis, we plotted the significant during temperature- and CabTRP Ia-mediated rescue, respectively. For the temperature rescue, we heated STG and CoGs together to crash temperature to rescue the rhythm (similar to Figure 4A). For the CabTRP Ia rescue, we heated only the STG to crash temperature and then applied CabTRP Ia selectively to the STG to rescue the rhythm (similar to Figure 6). For both rescue conditions, the significant vectors were statistically different from equal distribution (Figure 7D), and *z* values were high (temperature rescue: 24.3, CabTRP Ia rescue: 47.2), indicating that integer coupling was present. We also found that the phase at which the LG started within the pyloric cycle was statistically different from a random distribution for both rescue conditions (Figure 7E) with high *z* values (temperature rescue: 38.5, CabTRP Ia rescue: 48.9). Since neither heating the CoGs nor applying CabTRP Ia altered the coupling mechanism between the rhythms, our data further verifies that integer coupling is intrinsic to the STG circuits.

### Integer Coupling Becomes Weaker With Increasing Temperature

We noted that the significant in the temperature rescue experiments, while significant, had a wider range than at most pre-crash temperatures and concurrently lower *z*-values in the Rayleigh analysis. This was surprising since we expected temperature rescued rhythms after warming STG and CoGs together to show a robust integer coupling, as suggested by Powell et al. (2021). To resolve this mystery, we looked at individual experiments rather than the averaged data. Figure 7F (left) shows original recordings of a temperature-rescued gastric mill rhythm at 19°C, and the simultaneously recorded pyloric activity (PD). The colors indicate the timing of the LG burst within the pyloric cycle, i.e., its onset phase. Unexpectedly, LG’s onset phase was not constant, although the rhythm was regular and long-lasting. For example, the LG burst began at phase 0.65 for the first LG burst (top recordings), while the LG burst of the subsequent cycle (second recording from top) started at phase 0.18, suggesting that the two rhythms were not tightly coupled anymore. In contrast to this experiment, we also found examples where LG’s onset phase mainly remained constant in the rescued rhythm (Figure 7F, right). Thus, there appeared to exist at least two data populations, one where strong integer coupling was maintained at higher temperature and another one where integer coupling was mostly absent despite regular rhythmic activity.

To exclude that the observed shift in phase-relationship in some preparations was not an idiosyncrasy of our specific approach where just parts of the preparation were heated, we compared our data to the publicly available data set from Powell et al. (2021), where the whole preparation was heated uniformly. We re-plotted the significant from the Powell et al. (2021) data set in rose diagrams (Supplementary Figure 1A). In addition, we analyzed the phase relationship between PD and LG onset and calculated the phase vectors (Supplementary Figure 1B). To our surprise, we found very similar results to our experiments. At all temperatures, the significant and phase vectors in the Powell et al. data set were significantly different from random distributions, demonstrating that, on average, integer coupling was present. However, we also found that with increasing temperature, the range of significant and phases broadened.

Furthermore, when we looked at individual preparations, we found that some showed varying LG onset phases and increasingly so at higher temperatures, while others maintained a stable onset phase. Supplementary Figure 1C shows an example of one such experiment. The onset phase of the LG burst varied dramatically, from 0.23 (top recording) to 0.76 during the subsequent gastric mill cycle (second from top). Consequently, even when the whole nervous system is heated uniformly, two subpopulations of responses seem to be present.

These results thus demonstrate that even in small circuits, idiosyncrasies exist between individuals, resulting in distinct responses to temperature challenges. This fits well with previously suggested variabilities between individuals concerning varying ion channel expression levels (Schulz et al., 2006; Goaillard et al., 2009) and responses to perturbations and physical variables such as pH and temperature (Alonso and Marder, 2020; He et al., 2020), and the general idea of degenerative circuits (Powell et al., 2021).

## Discussion

### Is the Gastric Mill Rhythm Temperature-Robust or Temperature-Sensitive?

In contrast to the pyloric rhythm that has long been described as robust over a broad range of temperatures (Tang et al., 2010; Soofi et al., 2014), the gastric mill rhythm’s temperature response has been somewhat enigmatic. The reason for this lies within the gastric mill rhythm’s episodic character. It is only intermittently active when food is present (or the equivalent sensory pathways are activated) and can thus not be recorded continuously over long periods. It is also more challenging to separate potential temperature effects on the gastric mill circuit from those on the sensory pathways that activate it. Finally, the gastric mill pattern shows substantial variability in its burst properties and cycle period, which makes it more challenging to determine crash points.

The gastric mill network is distributed over several ganglia and includes descending projection neurons in the commissural ganglia and the CPG circuit neurons in the STG. The projection neurons innervate the CPG neurons and are required to activate the rhythm (Nusbaum and Beenhakker, 2002; Stein, 2009, 2017). Projection neurons and central pattern generating neurons can be separately exposed to varying temperatures. This allows temperature effects on rhythm-driving neurons to be dissociated from those on the central pattern generating circuit. Powell et al. (2021) demonstrated that the VCN gastric mill rhythm is similarly temperature-robust as the pyloric rhythm when both the descending projection neurons and the central pattern generating neurons are heated together. Here in our study, we are providing the mechanism for this temperature robustness by showing that the VCN gastric mill rhythm is maintained over a broad temperature range because of an increase in projection neuron activity - specifically that of MCN1. The STG central pattern generating network itself is temperature sensitive. In a previous study, we showed that leak conductance of the core gastric mill CPG neuron LG increases with temperature (Städele et al., 2015). If not counterbalanced by MCN1, this increase in leak conductance shunts synaptic signal propagation and stops LG spike activity and consequently the gastric mill rhythm (DeMaegd and Stein, 2021). While in this previous study the gastric mill rhythm only involved one projection neuron, namely MCN1, it is likely that a similar increase in LG leak conductance occurs in all versions of the gastric mill rhythm when temperature is raised at the STG circuits.

MCN1 releases the substance P-related neuropeptide, CabTRP Ia (*Cancer borealis* tachykinin-related peptide Ia), and our previous findings show that CabTRP Ia can counterbalance the temperature-induced increase in LG’s leak and sustain LG activity at elevated temperature when bath-applied or neuronally released. This temperature compensation is mediated through the neuromodulator-induced current I_MI_ (DeMaegd and Stein, 2021), which is activated by CabTRP Ia and several other neuropeptides, as well as some muscarinic agonists (Swensen and Marder, 2000). Crustacean cardioactive peptide (CCAP), for example, is a hormone that induces I_MI_ in LG (DeLong et al., 2009) and can also confer temperature robustness to the gastric mill rhythm (DeMaegd and Stein, 2021). It is thus conceivable that extrinsic neuropeptide modulation sustains all gastric mill rhythms, even those independent of MCN1, and is likely to bestow considerable temperature robustness to the gastric mill rhythm *in vivo*.

Similar rescue mechanisms may also contribute to the robustness (or lack thereof) in other circuits and animals. Independently of the specific circuit, elevated temperature will in almost all cases lead to an increase of ionic conductance and leak currents. Membrane shunting because of increased leak currents has long been known to play essential roles in the regulation of excitability (Rekling et al., 2000; Brickley et al., 2007), switching neuronal activity states (Gramoll et al., 1994), and in the control of network oscillations (Cymbalyuk et al., 2002; Blethyn et al., 2006; Koizumi et al., 2008; Zhao et al., 2010). Consequently, modulation of leak currents by neurotransmitters has been proposed to contribute to the regulation of neuronal excitability (Aller et al., 2005; Kim, 2005; Pratt and Aizenman, 2007). There are also several examples where neuromodulators, and neuropeptides in particular, have been implicated in supporting robust activity in several pattern-generating networks (Gray et al., 1999; Mutolo et al., 2010; Zhao et al., 2011; Zhu et al., 2018; Ellingson et al., 2021; Shi et al., 2021). Increasing the robustness of a circuit through extrinsic modulation may be especially meaningful when perturbations occur rapidly, like in intertidal species that experience substantial temperature variations within a few minutes (Stein and Harzsch, 2021).

Interestingly, our data also suggests that some aspects of the temperature response of the pyloric rhythm are likely to be mediated by extrinsic modulation. Specifically, the heat-induced increase in the cycle frequency of the pyloric rhythm was reduced without heating of the commissural ganglia (Figure 3D). This indicates that the speeding up of the pyloric rhythm observed in intact animals (Soofi et al., 2014) and during heating of the entire nervous system (Tang et al., 2010, 2012; Haddad and Marder, 2018; Powell et al., 2021) is likely influenced by factors extrinsic to the pyloric CPG.

### Factors That Determine the Temperature Response of the Gastric Mill Rhythm

Separating the effects of temperature on the various neuronal players involved in eliciting and maintaining the gastric mill rhythm is challenging. Our results show that the gastric mill CPG has very little intrinsic ability to compensate the effects of increased temperatures. Instead, our data suggests that extrinsic modulation provided by MCN1 is a crucial factor that enables the gastric mill rhythm to continue to function at elevated temperature. It remains unknown, however, why MCN1 activity increases with temperature. The principal options are that more or stronger synaptic inputs are present, or that the intrinsic excitability of MCN1 increases. VCN stimulation inhibits MCN1. With repeated stimulus trains MCN1 rebounds from inhibition and enters a long-lasting phase of bursting (Beenhakker et al., 2004) which ultimately elicits the VCN gastric mill rhythm. Higher temperatures are likely to increase both the synaptic inhibition to MCN1 and the rebound from it, thus leading to increased MCN1 activity. Alternatively, additionally recruited synaptic inputs could contribute to an enhanced inhibition.

In our experiments, we extracellularly stimulated the axons of the VCN neurons in the *dpon*, which excludes additional recruitment of synaptic inputs. There are about 60 VCN neurons on each side of the cardiac gutter. While the exact number of VCN axons that project through the *dpon* is unknown, previous studies have shown that changes in stimulation strength can recruit different numbers of VCN axons (Beenhakker et al., 2004). Temperature perturbations can dramatically alter axonal rheobase (Guttman, 1968), affecting how effective a stimulus can recruit axons. For example, a temperature increase could decrease the rheobase and increase the number of recruited VCN axons. In that case, MCN1 would receive more synaptic inhibition, which potentially increases the rebound and the level of excitation MCN1 provides to the gastric mill CPG. However, in our experiments, we prevented these confounding effects of the temperature changes on the stimulation site by keeping the stimulation site in a separate temperature compartment, with constant temperature throughout the experiment. It is thus unlikely that temperature effects on the stimulation affected the outcome of our investigations.

Increased release from projection neuron terminals in the STG may also play a role in rescuing the gastric mill rhythm from a temperature-induced crash. Transmitter release, and in particular neuropeptide release, has been shown to be temperature-dependent in other invertebrate systems, such as Aplysia (Vilim et al., 1996), even when presynaptic spike activity remains the same. While it is likely that peptide release from MCN1 terminals changes with temperature, our data indicate that a heat-induced change in synaptic release is not a deciding factor for rescuing the gastric mill rhythm at elevated temperature. While the regulation of the activity of the modulatory projection neurons is complex, we find that projection neuron activity does not change when only the STG is heated (Figure 5). There is also no rescue of the rhythm despite a potential change in release from the terminals in that condition.

In contrast, when STG and CoGs are heated together, projection neuron firing frequency increases, and the rhythm is rescued. This is consistent with our previous findings that constant projection neuron firing frequency is insufficient to rescue the rhythm from a heat crash (Städele et al., 2015), even if projection neuron transmitter release may increase. Instead, higher firing frequencies are required. The conclusion must be that while MCN1’s release properties very likely do change with temperature, its firing frequency (and the subsequent increase in transmitter release) is critical for the rescue of the rhythm.

### Gastro-Pyloric Integer Coupling Is Temperature-Robust and Arises From Network-Intrinsic Mechanisms

The integer coupling between the pyloric and gastric mill rhythms has been studied in detail for one version of the gastric mill rhythm, namely the one elicited by tonic stimulation of MCN1 (Nadim et al., 1998). Integer coupling is due to two synaptic connections. The gastric mill CPG creates antiphase bursting through a ‘release’ mechanism (Skinner et al., 1994), i.e., LG can only burst when it is released from the synaptic inhibition it receives from its half-center antagonist Interneuron 1. The LG bursts thus always occurs in the gaps between the interneuron 1 bursts. These gaps are timed to the pyloric rhythm because they occur whenever interneuron 1 is inhibited by the pyloric pacemakers (specifically by the anterior burster AB, which is the main pacemaker neuron driving the pyloric rhythm). This results in a disinhibition of LG. As a consequence, the LG burst only starts whenever AB is bursting. Pyloric and gastric mill rhythms are thus linked, with the gastric mill cycle period being an integer multiple of the pyloric period.

Whether the same mechanisms of gastro-pyloric coupling are present and equally important in all versions of the gastric mill rhythm is not clear. It is at least conceivable that other influences that contribute to generating the gastric mill rhythm may dilute the effects of the disinhibition on LG and allow LG to burst at times not fixed to the pyloric rhythm. However, integer coupling has been demonstrated in at least three additional versions of the gastric mill rhythm: (i) one elicited pharmacologically by the presence of pyrokinin peptide (Saideman et al., 2007), (II) one elicited by stimulation of a peptidergic pathway (Blitz et al., 2008), and (iii) the one elicited by VCN stimulation (Powell et al., 2021). The data from our study further suggests that gastro-pyloric integer coupling is independent of changes to extrinsic modulation, and thus likely intrinsic to the CPG circuits and synaptic connections within the STG. With respect to temperature influences, we found that independently of whether only the STG or the whole nervous system was heated, integer coupling was present. In fact, whenever the gastric mill rhythm was active, it was integer coupled with the pyloric rhythm. Temperature changes did not disrupt the integer coupling, further suggesting that the two synapse-connection between the pyloric pacemaker AB and LG (see Figures 1B, 7A, inset) is temperature-resilient and remains active over a broad temperature range.

However, our results demonstrate that integer coupling becomes more variable and unstable when approaching gastric mill crash temperature. Specifically, while the LG burst still started during the interburst of Interneuron 1, the delay with which the burst started could vary substantially. Thus, inducing LG bursts still required disinhibition from Interneuron 1, but recovering from this inhibition became more variable, making the coupling between the rhythms less stringent. A delayed recovery could result from LG being less likely to produce action potentials as temperatures increase and cycle periods increase (Figure 2). This is consistent with an increase in rheobase observed previously when temperature increases (Städele et al., 2015). LG could thus be more prone to fail to properly initiate a burst, resulting in varying delays between the pyloric disinhibition and the LG burst onset.

### Future Studies

The pyloric and gastric mill rhythms function over a similar temperature range. The mechanisms that support their temperature-robust activity, however, differ substantially. The pyloric rhythm is pacemaker-driven and continuously active throughout the animal’s life span. Maintaining a balance of ionic conductances that enables the pacemaker to oscillate is thus critical to ensure continuous functioning when temperatures change (O’Leary and Marder, 2016; Alonso and Marder, 2020).

In contrast, the gastric mill rhythm is episodic, requires extrinsic excitation to be activated, and its rhythmicity stems from the synaptic interactions between the gastric mill CPG neurons. Network oscillators like the gastric mill circuit generate rhythmic activity as long as individual neurons produce action potentials and the synaptic strength is high enough. This is particularly the case for networks with successive inhibitory synaptic connections (like ring oscillators with odd numbers of neurons). Individual neurons do not necessarily need to possess properly tuned intrinsic properties to enable rhythmic bursting. Temperature-robust activity, in this case, requires mainly the maintenance of the synaptic interactions and the ability to continuously produce action potentials. The requirement for matching intrinsic ionic conductances, in contrast, may be less stringent. The mechanism that enables temperature-robust activity in the gastric mill circuit fits these expectations. An increased peptide transmitter release from MCN1 counterbalances the temperature-induced shunt in LG, enabling it to continue to produce action potentials (Städele et al., 2015; DeMaegd and Stein, 2021). This mechanism may also provide more flexibility to the temperature responses, as it can be continuously and rapidly adapted through changes in the firing frequency of the peptide releasing neurons.

The mechanisms that enable temperature robustness in the pyloric and gastric mill rhythms are not mutually exclusive since neither circuit is purely pacemaker- or network-driven. For example, the pyloric pacemakers receive synaptic feedback from a follower neuron called LP. This feedback, while not crucial for generating the rhythm, contributes to maintaining rhythm speed (Thirumalai et al., 2006). The pyloric circuit is also affected by the same extrinsic modulation as the gastric mill circuit. For example, cutting all extrinsic inputs to the STG dramatically slows down the pyloric rhythm, and sometimes even completely stops it (Khorkova and Golowasch, 2007). In our experiments, the heat-induced speed-up of the pyloric rhythm was lessened when extrinsic modulatory pathways were not heated. Likewise, some gastric mill neurons possess pacemaker-like intrinsic properties that support bursting, such as plateau potentials (Katz and Harris-Warrick, 1989). Thus, it is likely that there are multiple mechanisms for temperature compensation that complement each other.

Future experimentation is necessary to determine the weighting and impact of these mechanisms in each circuit. Models have predicted that ionic conductances change appropriately with temperature to enable the temperature robustness of the pyloric rhythm. This idea is still lacking experimental evidence. Further, it is unclear if and how these mechanisms enable individuals or even whole species to combat temperature changes in their habitats. This is a particularly interesting aspect given the continuous warming of the atmosphere and the oceans due to global climate change, and the ever-increasing occurrence of temperature extremes. However, for such studies, comparisons of animals from the same species but different temperature habitats are required. In addition, it can be expected that species that live in habitats that experience few temperature fluctuations (such as the deep sea) show different temperature responses and compensation capabilities than animals directly exposed to large temperature fluctuations. Here, comparisons across species, but using the homolog neuronal circuits (such as the pyloric and gastric mill circuits) would offer significant insight. There are already first indications that homeostatic or evolutionary processes can adjust the operational temperature range of a circuit. For example, the maximum temperature at which the pyloric rhythm functions is correlated with the average water temperature the animal experienced in the year it was caught (Marder and Rue, 2021).

Concerning temperature responses and the effectivity of the mechanisms that counterbalance temperature effects, it is also necessary to test their state-dependence. This includes testing variations in responses and compensation mechanisms during seasonal changes, lunar and reproductive cycles, different times of day, and between male and females. No studies to date, including this one, has addressed these influences. Finally, the mere existence of a pattern does not necessarily mean that a behavior can be carried out. The temperature responses of the muscles that carry out the behavior must also be studied to address the question if changes to the produced patterns that are caused by temperature fluctuations are adequately received and carried out. This includes changes in rhythm speed, firing frequencies, and burst properties. We already know that even small changes in LG burst activity, for example, are associated with changes in the movement of the stomach teeth (Heinzel, 1988a,b; Diehl et al., 2013), suggesting that the temperature-induced changes in the LG duty cycle we measured at higher temperatures will lead to changes in the behavioral output.

## Data Availability Statement

The raw data supporting the conclusions of this article will be made available by the authors, without undue reservation.

## Author Contributions

CS: conceptualization, data curation, software, investigation, visualization, methodology, writing – original draft, and writing – review and editing. WS: conceptualization, visualization, supervision, funding acquisition, validation, project administration, writing – original draft, and writing – review and editing. Both authors: contributed to the article and approved the submitted version.

## Conflict of Interest

The authors declare that the research was conducted in the absence of any commercial or financial relationships that could be construed as a potential conflict of interest.

## Publisher’s Note

All claims expressed in this article are solely those of the authors and do not necessarily represent those of their affiliated organizations, or those of the publisher, the editors and the reviewers. Any product that may be evaluated in this article, or claim that may be made by its manufacturer, is not guaranteed or endorsed by the publisher.
